# EGFR in enterocytes & endothelium and HIF1α in enterocytes are dispensable for massive small bowel resection induced angiogenesis

**DOI:** 10.1371/journal.pone.0236964

**Published:** 2020-09-15

**Authors:** Emily J. Onufer, Bola Aladegbami, Toru Imai, Kristen Seiler, Adam Bajinting, Cathleen Courtney, Stephanie Sutton, Aiza Bustos, Junjie Yao, Cheng-Hung Yeh, Anne Sescleifer, Lihong V. Wang, Jun Guo, Brad W. Warner

**Affiliations:** 1 Division of Pediatric Surgery, Department of Surgery, St. Louis Children’s Hospital, Washington University in St. Louis School of Medicine, St. Louis, MO, United States of America; 2 Department of Biomedical Engineering, Washington University in St. Louis, St. Louis, MO, United States of America; 3 Department of Electrical Engineering, Caltech Optical Imaging Laboratory, Andrew and Peggy Cherng Department of Medical Engineering, California Institute of Technology, Pasadena, CA, United States of America; 4 Saint Louis University School of Medicine, St. Louis, MO, United States of America; University of Edinburgh, UNITED KINGDOM

## Abstract

**Background:**

Short bowel syndrome (SBS) results from significant loss of small intestinal length. In response to this loss, adaptation occurs, with Epidermal Growth Factor Receptor (EGFR) being a key driver. Besides enhanced enterocyte proliferation, we have revealed that adaptation is associated with angiogenesis. Further, we have found that small bowel resection (SBR) is associated with diminished oxygen delivery and elevated levels of hypoxia-inducible factor 1-alpha (HIF1α).

**Methods:**

We ablated EGFR in the epithelium and endothelium as well as HIF1α in the epithelium, ostensibly the most hypoxic element. Using these mice, we determined the effects of these genetic manipulations on intestinal blood flow after SBR using photoacoustic microscopy (PAM), intestinal adaptation and angiogenic responses. Then, given that endothelial cells require a stromal support cell for efficient vascularization, we ablated EGFR expression in intestinal subepithelial myofibroblasts (ISEMFs) to determine its effects on angiogenesis in a microfluidic model of human small intestine.

**Results:**

Despite immediate increased demand in oxygen extraction fraction measured by PAM in all mouse lines, were no differences in enterocyte and endothelial cell EGFR knockouts or enterocyte HIF1α knockouts by POD3. Submucosal capillary density was also unchanged by POD7 in all mouse lines. Additionally, EGFR silencing in ISEMFs did not impact vascular network development in a microfluidic device of human small intestine.

**Conclusions:**

Overall, despite the importance of EGFR in facilitating intestinal adaptation after SBR, it had no impact on angiogenesis in three cell types–enterocytes, endothelial cells, and ISEMFs. Epithelial ablation of HIF1α also had no impact on angiogenesis in the setting of SBS.

## Introduction

Short bowel syndrome (SBS) results from extensive surgical loss of small intestine (SI), resulting in severely diminished absorptive capacity and the inability to meet nutritional demands. Incidence of SBS drastically increases in preterm infants compared to full term infants, reported at 353.7 compared to 3.5 per 100,000 live births, respectively[1]. In addition to a 27% mortality rate, SBS has significant morbidity, associated with the need for prolonged parenteral nutrition leading to catheter-associated sepsis and intestinal failure associated liver disease[2–5].

Following massive small bowel resection (SBR), the remaining SI undergoes adaptation, a critical compensatory process characterized by increased enterocyte proliferation leading to villus lengthening and crypt deepening. This expands mucosal surface area for nutrient absorption[6, 7]. If the remnant SI fails to adapt, patients require parenteral nutrition, which is associated with its own added morbidity[8].

In addition to enhanced enterocyte proliferation, intestinal adaptation after SBR is associated with new blood vessel growth (angiogenesis) within the intestinal villi and submucosal layers[9, 10]. This proliferation of new vessels from a preexisting vascular bed allows for enhanced influx of nutrients, growth factors, oxygen, and immune cells, to stimulate repair and growth[11, 12]. Resection-associated angiogenesis may play a role in maintaining mucosal integrity and increasing absorptive capacity, both of which are important in the setting of SBS. Additionally, increased microvascular flow may enhance absorptive capacity in patients with SBS[13]. On the other hand, villus hypoperfusion is associated with disrupted mucosal integrity and sets the stage for bacterial translocation and sepsis[14, 15].

Using photoacoustic microscopy (PAM), we previously identified a significant fall in oxygen saturation and blood flow within the remnant SI within minutes after SBR *in viv*o. This suggests that resection-associated angiogenesis may be triggered by an initial hypoxic signal[16]. Hypoxia-inducible factor 1-alpha (HIF1α) is a major mediator of hypoxia-induced angiogenesis, and has been shown to be elevated following SBR[17]. HIF1α is stimulated not only by hypoxia, but also by proinflammatory microenvironments, and by stimulus of certain growth factor receptors[18]. One example of this is Epidermal Growth Factor Receptor (EGFR)[19–21]. A link between EGFR and HIF1α signaling has been established in a prototypical intestinal epithelial cell line, with Epidermal Growth Factor (EGF) providing HIF1α stabilization[22]. And, whereas EGFR can induce the accumulation of HIF, HIF signaling has also been shown to activate EGFR signaling[23, 24].

Following SBR, there is a significant increase in the expression and activity of intestinal EGFR (including epithelial and subepithelial EGFR), which has been shown to contribute to adaptation. Interestingly, however, epithelial-specific EGFR was found to be dispensable for structural adaptation[25–28]. In that study, we did not examine the angiogenic response to epithelial EGFR knockout. Our prior findings did lead us to speculate that EGFR signaling in the subepithelium may be just as if not *more* critical than epithelial EGFR signaling during adaptation. As such, our hypothesis for this study is that subepithelial EGFR signaling drives resection-associated angiogenesis.

The subepithelium contains numerous cell types including nerves, blood vessels with associated endothelial cells (ECs), lymphatics, immune cells, and stromal cells. Unlike intestinal epithelium (unique in its expression of the Villin promotor), organ-specific knockouts of these tissue components are not feasible. Rather, any gene knockout in these tissue types would be global. Therefore, to test our hypothesis, we elected to focus on vascular ECs. We chose ECs because they secrete angiocrine factors which support and sustain homeostasis and regeneration of non-endothelial stem and progenitor cells after tissue injury and during repair[29]. This should ostensibly include adaptation to SBR, an assumption supported by the fact that EC-derived angiocrine signals are necessary for resection-associated regeneration of the liver and lung[30, 31].

No prior studies have tested whether EGFR signaling in vascular ECs significantly contributes to resection-induced adaptation and/or angiogenesis. Given that intestinal hypoxia is associated with massive SBR, the aim of this study was to determine the contribution of epithelial as well as endothelial EGFR and HIF1α activity and expression to structural adaptation, angiogenesis, and regulation of resection-induced blood flow to the remnant bowel.

## Materials and methods

We believed that EGFR is essential for angiogenesis and, therefore, ablated it in the epithelium and endothelium using Villin-cre^ER^ and Tie2-cre^ER^ transgenic mice crossed with an EGFR (flox/flox) line. Given the role of HIF1α in angiogenesis, we also ablated it the epithelium, ostensibly the most hypoxic element. Using these mice, we first determined the effects of these genetic manipulations on intestinal blood flow after SBR. In another set of experiments, we determined the effect of these genetic manipulations on intestinal adaptation and angiogenic responses. Then, given that ECs require a stromal support cell for efficient vascularization, we ablated EGFR expression in intestinal subepithelial myofibroblasts using lentiviral silencing to determine its effects on vascularization in a microfluidic model of human small intestine[32].

### Animals

The Washington University Animal Studies Committee, which is an animal ethics committee that consists of experts at our institution, reviewed and approved the protocol for this study (Protocol #20170252) in accordance with the National Institute of Health and laboratory animal care use and guidelines. All mice underwent a proximal SBR, as described below. C57BL/6J and *Hypoxia-Inducible Factor 1-alpha (HIF1α)(flox/flox)* mice were purchased from the Jackson laboratory, while Epidermal growth factor receptor-floxed (*Egfr*^*tm1Dwt*^) mice were generously provided by David Threadgill (University of North Carolina, Chapel Hill). *Villin Cre*^*ER*^ mice were obtained via a generous donation from Dr. Sylvie Robine (Curie Institute, Paris, France) and *Tie-2-Cre*^*ER*^ mice were created by *in vitro* fertilization of C57BL/6J (Washington University Mouse Genetics Core, DDRCC, St. Louis, MO). Intestinal epithelial-specific knockout (KO) mice were generated by crossing *Villin Cre*^*ER*^ mice with *Epithelial Growth Factor Receptor (EGFR)(flox/flox)* and *Hypoxia-Inducible Factor 1-alpha (HIF1α)(flox/flox)*. While endothelial specific KO mice were generated by crossing *Tie-2-Cre*^*ER*^ mice with *Epithelial Growth Factor Receptor (EGFR)(flox/flox)*.

Wild type (WT) littermates *Villin Cre*^*ER*^(-) or *Tie-2-cre*^*ER*^(-) and C57BL/6J were used as controls. Mutant mice were maintained on a C57BL/6J background. Both male and female mice were used for SBR experiments. Tamoxifen (Sigma, St. Louis MO) was injected at 1.25mg/25g body weight for 3 consecutive days to induce deletion of gene expression in the intestinal epithelial specific-KO mice (*Villin Cre*^*ER*^), while the endothelial specific-KO mice (*Tie-2-cre*^*ER*^) received 3.75mg/25g bodyweight for 7 consecutive days[33]. Mice were kept in the animal holding area with a 12-hour light-dark cycle and given rodent chow ad lib after weaning. Mice were then given a liquid rodent diet (Micro-Stabilized Rodent Liquid Diet LD101; Purina Mills, St. Louis, MO) 1–2 days prior to surgery. The average weight for each mouse line at operation for the immunohistochemistry experiments was obtained (*VillinCre*^*ER*^*(+/-)HIF1*α*(flox/flox)* WT-21.3gm, KO-19.4gm; *VillinCre*^*ER*^*(+/-)EGFR(flox/flox)* WT-21.3gm, KO-19.0gm; *Tie2Cre*^*ER*^*(+/-)EGFR(flox/flox)* WT-20.6gm, KO-18.5gm). At harvest, mice were euthanatized using a ketamine injection with cervical dislocation.

### Operative technique

Mice aged 8–10 weeks underwent 50% proximal SBR as we have previously described[34]. Anesthesia was provided using 0.25% Marcaine injection (2mg/kg) subcutaneously. Briefly, mice underwent small bowel transection at a point 12cm proximal to the cecum and also at a point 1-2cm distal to the ligament of Treitz. The mesentery was ligated with 3–0 silk and the intervening bowel was removed. Intestinal continuity was achieved by re-anastomosis of the bowel with 9–0 monofilament suture. In the immediate 24-hour post-operative period, the mice were given water ad libitum. They were then restarted on liquid rodent diet. Mice were monitored every 12 hours for two days postoperatively and then daily after by assessing water and food intake as well as physical signs of distress.

### Confirmation of enterocyte and endothelial cell gene knockout

Small intestine mucosa was first isolated from *Villin Cre*^*ER*^*(+/-)EGFR* and *Villin Cre*^*ER*^*(+/-)HIF1α* mouse lines as previously described[35]. EGFR and HIF1α deficiency were then confirmed via RT-PCR using total RNA extracted from enterocytes. For endothelial specific EGFR deficiency confirmation in the *Tie-2-CreER(+/-)EGFR(flox/flox)* mouse line, lung endothelial cells were used to verify the efficiency of gene ablation as previously described[36]. In brief, lung tissue was harvested and enzymatically digested (2mg/mL Collagenase I (Gibco 17100–017)) in Live Cell Imaging Solution (Invitrogen, A14291DJ) in a GentleMACS dissociator. Homogenates were filtered to remove undigested tissue and resuspended. Endothelial cells were then isolated from this lung cell suspension using CD146 MicroBeads (#130-092-007) and a MS Column (Miltenyi Biotec, Auburn, CA). Subsequently, total RNA was extracted from isolated endothelial cells and EGFR deficiency was verified via RT-PCR as detailed below.

### Measurement of structural adaptation

Formalin-fixed specimens of distal intestine (2cm in length, 2cm from anastomosis) were embedded into paraffin and then cut into longitudinal 5μm-sections. Villus height, the length from the tip of the villi to the crypt-villi junction, was measured using H&E stained sections by a single investigator blinded to mouse strain (NIS elements AR 4; Nikon, Melville, NY). A minimum of twenty crypts and villi were counted from 2cm intraoperative (IO) segments proximal to the anastomosis and compared with measurements from the distal remnant ileum on post-operative day (POD) 7. Adaptation was defined as a 15% increase in villus height and only mice that demonstrated adaptive villus growth were included in further analysis.

### Quantification of intestinal epithelial proliferation and submucosal capillary density

After tissue sections were formalin-fixed, paraffin-embedded, and sectioned, they were deparaffinized and blocked with 3% hydrogen peroxide in methanol. Antigen retrieval was performed using Diva Decloaking solution (Biocare Medical, Concord, CA) in a high-pressure cooker (120°C for 2 minutes). Slides were blocked with avidin-pink and biotin-blue (Biocare Medical, Concord, CA), and incubated with either anti-p-histone H3 antibody (#9701, 1:400; Cell Signaling Technology, Danvers MA) for proliferation or anti-CD31 antibody (#77699, 1:400; Cell Signaling Technology, Danvers MA) as a marker of angiogenesis in DaVinci Green (Biocare Technology, Concord, CA) overnight at 4°C. These were then visualized with biotinylated goat anti-rabbit IgG (Jackson ImmunoResearch Inc. West Grove, PA) followed by streptavidin-horseradish peroxidase (Jackson ImmunoResearch Inc. West Grove, PA), diaminobenzidine (Sigma-Aldrich, St. Louis, MO), and hematoxylin counterstaining.

As previously described, under light microscopy at 40x magnification, crypt enterocytes undergoing mitosis that were p-histone H3-stained were counted to assess for proliferation[37]. At least fifteen well-oriented fields of IO and POD7 samples per animal were counted by an investigator blinded to mouse strain. CD31-stained submucosal microvessels were also counted at 40x magnification to assess for blood vessel growth, as previously described[38]. At least ten well-oriented fields of IO and POD7 samples per animal were counted by an investigator blinded to mouse strain. Percentage change was calculated by matched mouse counts from IO to POD7.

### Intestinal sO_2_ and blood flow measurement by optical-resolution photoacoustic microscopy

**Fig 1:**
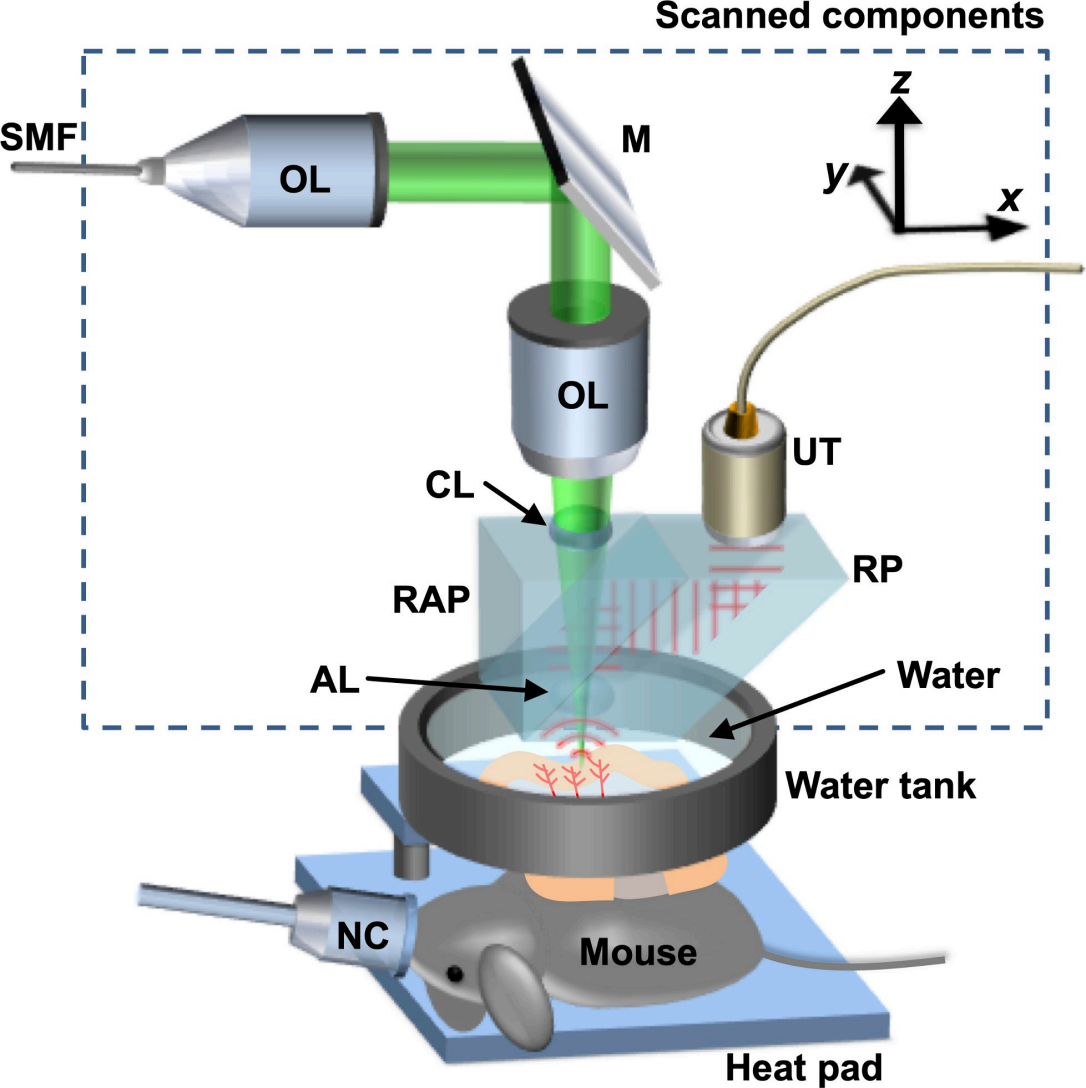
Schematic of optical-resolution photoacoustic microscopy (OR-PAM) for *in vivo* imaging of sO_2_ and flux of the intestinal blood vessels. AL, acoustic lens; CL, correction lens; M, mirror; NC, nose cone; OL, objective lens; RAP, right-angle prism; RP, rhomboid prism; SMF, single-mode fiber; UT, ultrasonic transducer.

PAM was performed prior to resection (pre-op), immediately after resection, and on POD3 as we have previously reported[16]. The variables measured included vessel diameter and density, blood flux, oxygen saturation of hemoglobin (sO_2_) within the terminal mesenteric arteriole and the accompanying vein at 6cm proximal to the ileocecal junction on the serosal surface of the intestine. To measure the sO_2_ level and flow of red blood cells (RBCs) in the intestinal blood vessels, we used our previously developed OR-PAM (Fig 1)[39].

Briefly, to calculate sO_2_, the system consisted of the two short pulse lasers to excite photoacoustic wave with two different wavelengths (InnoSlab, Edgewave, for 559 nm, and SPOT-10-200-532, Elfolight, for 532 nm). During imaging, each laser beam sequentially irradiated the target at 10 kHz. To detect the PA signal, the system employed a focused ultrasonic transducer with a 50 MHz central frequency (V214-BB-RM, Olympus). To acquire three-dimensional images, the system used two motorized translational stages, which enabled two-dimensional raster scanning. The step sizes for imaging were 2.5μm along the fast axis and 5μm along the slow axis. Imaging a 4mm by 4mm area took approximately 10 minutes. By taking advantage of the distinct difference of the optical absorption spectrum of the two forms of hemoglobin, oxygenated hemoglobin (HbO_2_) and deoxygenated hemoglobin (HbR), the relative concentration, hence sO_2_, can be calculated based on the PA signals acquired with at least two wavelengths[40]. In our system, wavelengths of 532nm and 559nm were used. To measure the flow of RBCs, the bandwidth broadening method was used[41, 42]. Using the wavelength of 532nm, 4000 A-lines were acquired at 10 kHz with M-Mode. By acquiring PA signals at multiple points across a blood vessel, a profile of the flow speed across the vessel as well as the vessel diameter can be measured. Based on the vessel diameter and the profile of the flow speed, the volumetric flow of blood per unit time (flux) can be calculated. Based on the measured two-dimensional sO_2_ images of the intestinal blood vessels, the oxygen extraction fraction (OEF) was calculated. A pair of primary artery and vein was first selected within the field of view, then, the average values of sO_2_ along the selected artery and vein were calculated. OEF was then calculated using the following equation: (*sO*_2_ *in artery* − *sO*_2_ *in vein*)/(*sO*_2_ *in artery*). During the measurement, the mice were under inhalational anesthesia with 1.5% isoflurane (Isothesia) and placed on a heating pad with a temperature control.

### Myofibroblast-derived EGFR effects on angiogenesis in a microfluidic model of human small intestine

#### Transwell and microfluidic co-culture

All experiments involving human tissues were carried out in accordance with protocols approved by the Washington University Human Research Protection Office (HRPO). Endothelial Colony Forming Cell-derived Endothelial Cells were isolated from de-identified samples of umbilical cord blood as previously described[43]. Human small intestine subepithelial myofibroblasts (ISEMFs) were isolated from fully deidentified surgical patients providing informed written consent, which is also witnessed and signed by the witness (Washington University Protocol 201504100, Dr. Deborah C. Rubin). All aspects of the study, including co-culture of multiple deidentified patient tissues, were performed according to Washington University protocol 201610114.

Co-culture experiments in transwell plates and microfluidic devices were performed as previously described[44]. After expansion and confirmation of EC and myofibroblast identities via RT-PCR, cells were co-cultured in a transwell or microfluidic device. Transwell co-cultures were performed using a 24-well polyester membrane insert with 0.4μm pore size (3450, Corning).

Microfluidic devices were fashioned as previously described[44]. In brief, devices were fabricated using a SYLGARD 184 Silicone Elastomer Kit (NC9285739, Dow Corning; Midland, MI) at a 10:1 PDMS curing agent ratio, plasma bonded to glass slides, and sterilized by autoclave and exposure to UV light overnight. The devices were then loaded with ECs and ISEMFs in a 1:1 ratio into the devices’ central chambers and media lines were loaded with EGM-2 media (CC-3162, Lonza; Basel, Switzerland). Media was changed daily. It has been previously confirmed that ECs alone do not form perfused capillary networks in the microfluidic device and that ISEMFs are part of the capillary network scaffolding[45]. All cultures were performed in a 5% oxygen, 5% CO2 incubator.

#### Lentiviral fluorescent protein transfections

In accordance with Washington University protocol (#2947), HEK293T was transduced using a three helper plasmid vectors (Addgene pMDLg-pRRE (Plasmid #12251), Addgene pRSV-Rev (Plasmid #12253), Addgene pMD2.G (Plasmid #12259)) and a third-generation lentiviral vector (Addgene pLV-Azurite, Plasmid #36086) to generate an azurite fluorescent protein lentiviral plasmid. Plasmid titers were added to ECs cultured in EGM-2 media with polybrene (5ug/ml) for 24–48 hours, with confirmation by fluorescent microscopy of a 50–90% stable transfection rate. Devices were imaged using inverted fluorescent microscopy (Olympus IX83 microscope, Olympus; Tokyo, Japan) with MetaMorph software (Molecular Devices; San Jose, CA).

#### Quantification of angiogenesis

Expansion of the of the fluorescent EC population was assessed in Image J (National Institutes of Health; Bethesda, MD). Number of junctions throughout the microfluidic device were manually counted per high power field. Vessel length is determined by junction to junction point, and was averaged over the microfluidic device (in μm per high power field).

#### EGFR silencing in intestinal subepithelial myofibroblasts

EGFR silencing in ISEMFs was accomplished using shRNA lentiviral particles and appropriate controls, per manufacturer protocol, in culture media supplemented with 5μg per ml polybrene (sc-44340-V, sc-108080, sc-108084, polybrene sc-134220, all Santa Cruz Biotechnology; Dallas, TX). EGFR mRNA and protein expression were tested one passage after the passage used in co-culture, confirming stability of the knockout for the duration of co-culture.

### RT-PCR

Isolated epithelial and endothelial cell RNA (RNeasy Mini Kit, Qiagen, Germantown, MD) and ISEMF RNA (Total RNA Purification Kit, 37500, Norgen, Thorold, ON) was extracted and its concentration determined using a NanoDrop Spectrophotometer (ND-1000; NanoDrop Technologies, Wilmington, DE). mRNA expression of EGFR (Hs01076090_m1, Applied Biosystems, Foster City, CA) and HIF1α (Mm01283757_m1, Applied Biosystems, Foster City, CA) were determined using Applied Biosystems 7500 Fast Real-Time PCR system with β-actin as the endogenous control (Mm04394036_g1, Applied Biosystems, Foster City, CA).

### Western blotting

After rinsing with Dulbecco’s Phosphate Buffered Saline (DPBS, 14040, Gibco), protein was isolated from cultured SIMF cells by lysing with sodium dodecyl sulfate sample buffer (50 mmol/L Tris-HCL, pH 6.8, 2% sodium dodecyl sulfate, 10% glycerol, and 5% 2-mercaptoethanol). Lysate was heated at 100°C and stored at -20°C prior to use. Protein concentration was measured using the RC DC (reducing agent and detergent compatible) Protein Assay Kit II (5000122, Bio-Rad; Hercules, CA). Proteins were loaded at equal concentrations, and detection was performed using the Bio-Rad ChemiDoc XRS+ system and image Lab software (Bio-Rad). Antibodies used were Anti-EGFR (06–847, 1:2500 Millipore; Burlington, MA), Anti-Phospho EGFR (2234, 1:1000, Cell Signaling Technologies; Danvers, MA), and Anti-Actin (4970S, Cell Signaling Technologies, 1:10000).

### Statistics

Statistical analysis was performed using GraphPad-Prism 7 software (La Jolla, CA). Differences were assessed using a two-way ANOVA with Tukey’s multiple comparison tests between groups with mouse lines or using the unpaired Student’s t test and one-way ANOVA for compiled PAM results. A p value of <0.05 was considered significant. Graphs with error bars represent mean + SEM.

## Results

### Confirmation of EGFR and HIF1α deficiency

**Fig 2:**
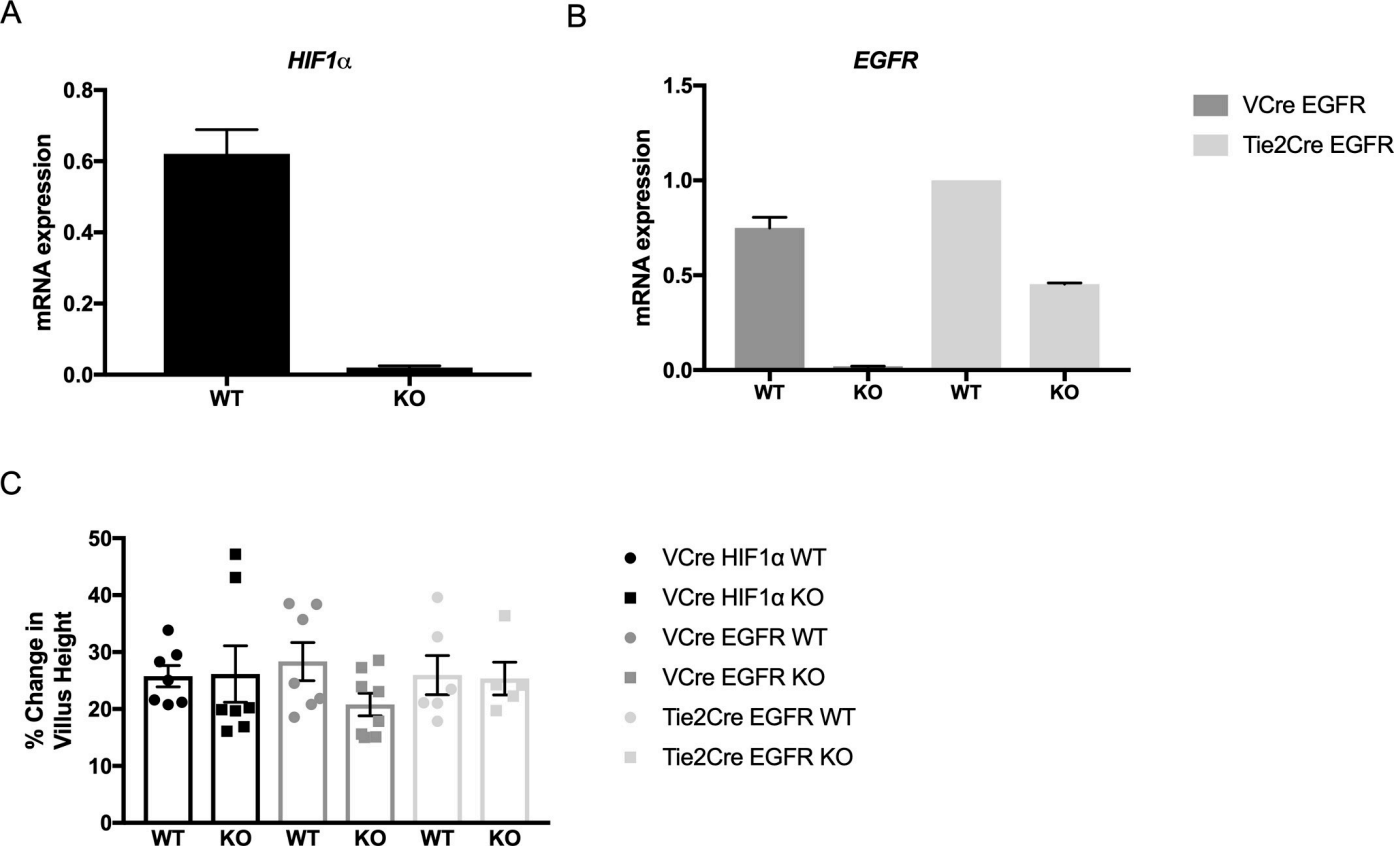
Knockout confirmation and structural adaptation. (A) Enterocyte HIF1α expression in *VillinCre*^*ER*^*(+/-)HIF1*α*(flox/flox)* WT (n = 7) and KO (n = 7) mice. (B) Enterocyte EGFR expression in *VillinCre*^*ER*^*(+/-)EGFR(flox/flox)* WT (n = 6) and KO (n = 8) mice and lung endothelial cell EGFR expression in *Tie2Cre*^*ER*^*(+/-)EGFR(flox/flox)* WT (n = 2) and KO (n = 2) mice. (C) Percent change in villus height from IO to POD7 in *VillinCre(+/-)HIF*α (WT n = 7, KO n = 7), *VillinCre*^*ER*^*(+/-)EGFR(flox/flox)* (WT n = 7, KO n = 8), and *Tie-2-Cre*^*ER*^*(+/-)EGFR(flox/flox)* (WT n = 6, KO n = 5) mouse lines after SBR.

The efficiency of EFGR and HIF1α ablation in enterocytes from mutant mice was confirmed with RT-PCR (Fig 2A and 2B). Similarly, RT-PCR of RNA isolated from endothelial cells in *Tie-2-Cre*^*ER*^*(+/-)EGFR(flox/flox)* confirmed EGFR mRNA was significantly decreased in the KO mice; however the deletion efficiency is not as high as in enterocytes, which may be due to different promoter activity or slower endothelium turnover rates (Fig 2B).

### Structural adaptation

Intestinal mucosal structural adaptation was demonstrated by percent change in villus growth from paired intraoperative controls. Equivalent degrees of morphological adaptation were observed between WT and KO mice after SBR in *VillinCre*^*ER*^*(+/-)HIF*α*(flox/flox)* (WT—25.8+/-1.9, KO -26.2+/-5.0, *VillinCre*^*ER*^*(+/-)EGFR(flox/flox)* (WT—28.3+/-3.3, KO—20.8+/-2.0), and *Tie-2-Cre*^*ER*^*(+/-)EGFR(flox/flox)* (WT—26.0+/-3.4, KO—25.4+/-2.9) mouse lines (Fig 2C).

### Intestinal epithelial proliferation

**Fig 3:**
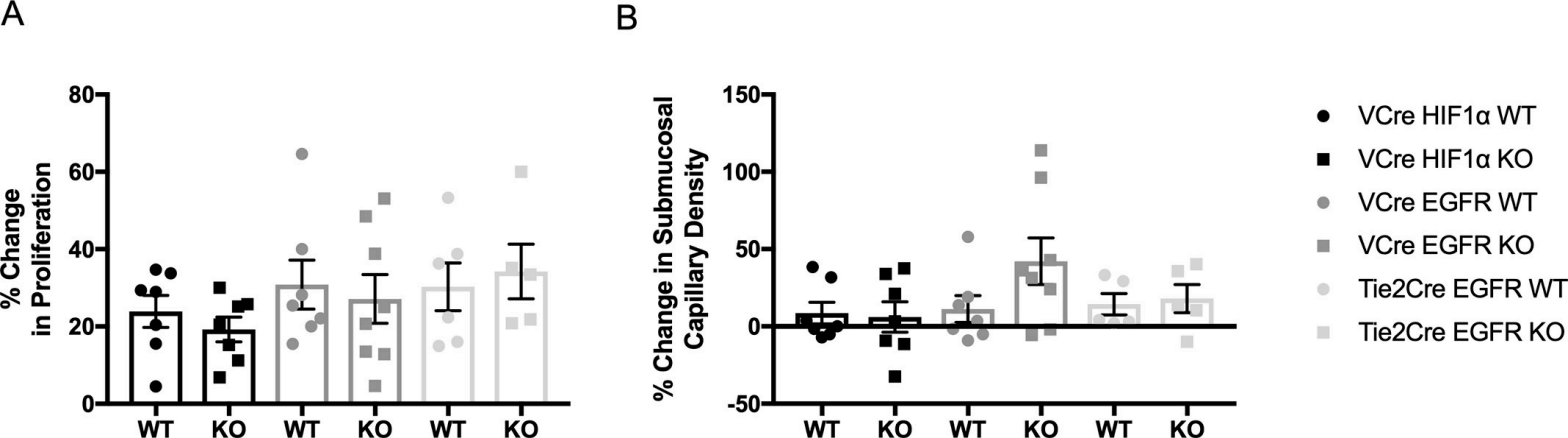
Changes in crypt proliferation and submucosal capillary density. (A) Percentage change in crypt proliferation from IO to POD7 in *VillinCre*^*ER*^*(+/-)HIF1*α*(flox/flox)* WT (n = 7) and KO (n = 7) mice, *VillinCre*^*ER*^*(+/-)EGFR(flox/flox)* WT (n = 7) and KO (n = 8) mice, and *Tie2Cre*^*ER*^*(+/-)EGFR(flox/flox)* WT (n = 6) and KO (n = 5) mice. (B) Percentage change in submucosal capillary density from IO to POD7 in *VillinCre*^*ER*^*(+/-)HIF1α(flox/flox)* WT (n = 7) and KO (n = 7) mice, *VillinCre*^*ER*^*(+/-)EGFR(flox/flox)* WT (n = 7) and KO (n = 8) mice, and *Tie2Cre*^*ER*^*(+/-)EGFR(flox/flox)* WT (n = 5) and KO (n = 5) mice.

Crypt proliferation was measured by immunostaining for phosphorylated histone H3. There were equivalent degrees of enhanced proliferation between WT and KO mice POD7 after SBR compared to matched control IO samples in *VillinCre*^*ER*^*(+/-)HIF*α*(flox/flox)* (WT—23.9+/-4.2, KO—19.2+/-3.2, *VillinCre*^*ER*^*(+/-)EGFR(flox/flox)* (WT—30.8+/-6.3, KO—27.1+/-6.2), and *Tie-2-Cre*^*ER*^*(+/-)EGFR(flox/flox)*(WT—30.3+/-6.1, KO—34.3+/-7.1) mouse lines (Fig 3A).

### Submucosal capillary density

The percentage change in submucosal capillary density was equivalent between WT and KO mice after SBR in *VillinCre*^*ER*^*(+/-)HIFα(flox/flox)* (WT—8.6+/-7.0, KO—6.1+/-9.8, *VillinCre*^*ER*^*(+/-)EGFR(flox/flox)* (WT—11.2+/-8.7, KO—42.12+/-15.1), and *Tie-2-Cre*^*ER*^*(+/-)EGFR(flox/flox)* (WT—14.4+/-7.0, KO—18.1+/-9.1) mouse lines (Fig 3B).

### Intestinal sO_2_ and blood flow measurement

**Fig 4:**
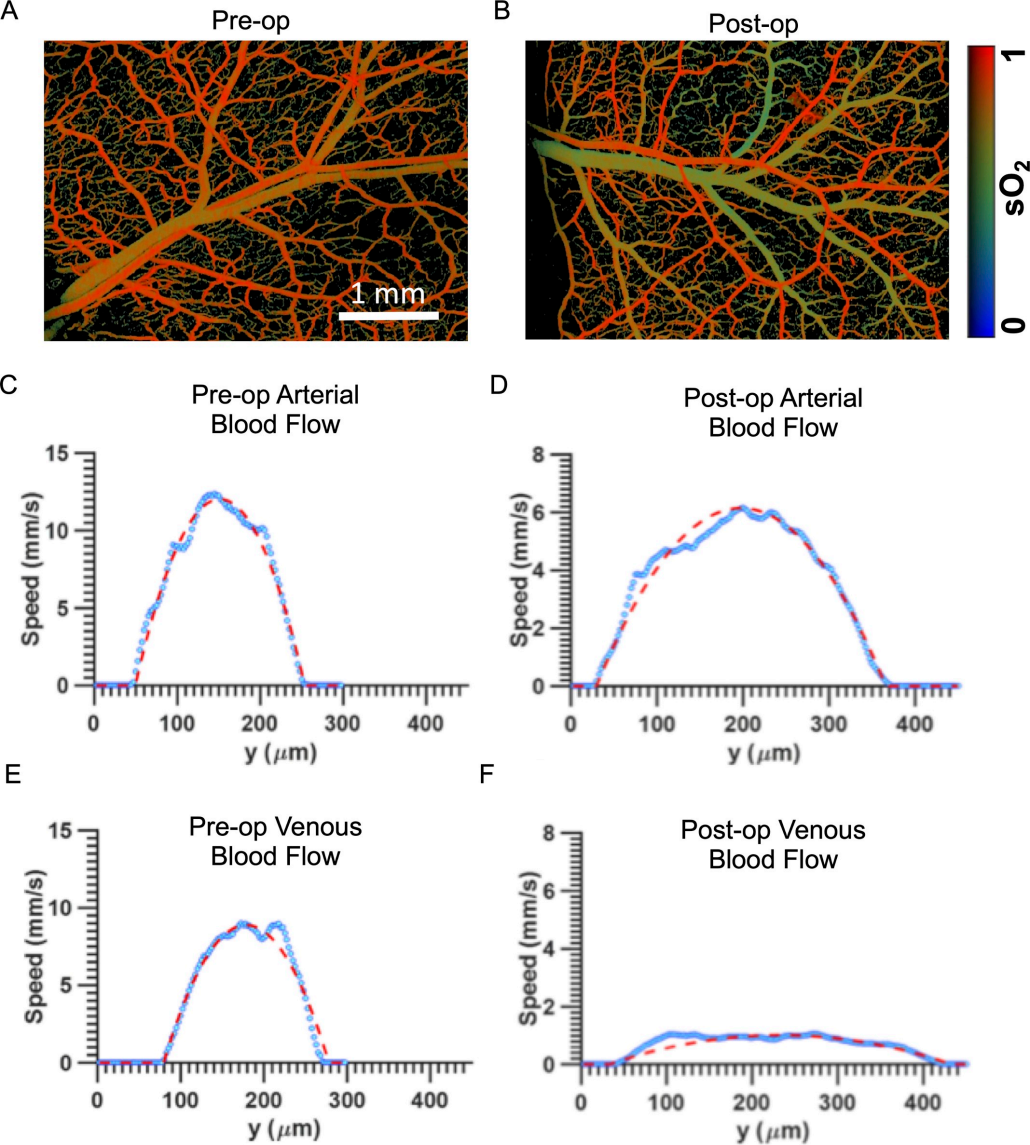
Representative sO_2_ images of intestinal blood vessels and blood flow profiles of the intestinal artery and vein. (A) sO_2_ image before SBR. (B) sO_2_ image after SBR. (C) Blood flow profile of the artery before SBR. (D) Blood flow profile of the vein before SBR. (E) Blood flow profile of the artery immediately after SBR. (F) Blood flow profile of the vein immediately after SBR. Blue circles represent the measured data, and the dashed red lines represent the fitting curve of the quadratic function in (C) to (F).

Fig 4A and 4B demonstrate representative 2D sO_2_ images, which are based on the PA images before and immediately after SBR. The sO_2_ in the main vein visually clearly dropped, suggesting a rapid immediate increase of the OEF.

**Fig 5:**
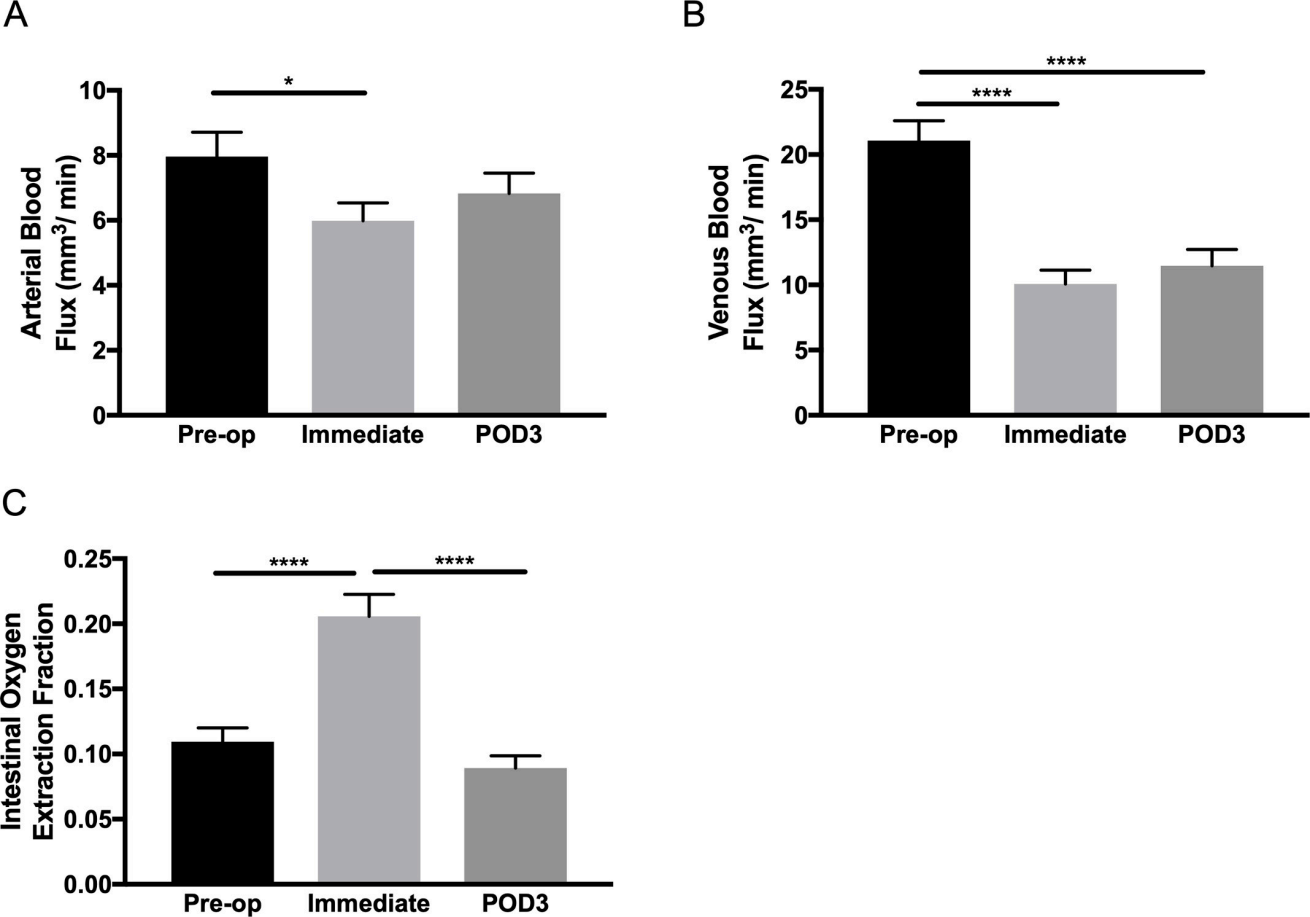
Ungrouped measurements of the hemodynamic response of OEF, with blood flow. (A) Ungrouped results of the arterial blood flow; *p<0.05, ANOVA ns. (B) Ungrouped results of the venous blood flow; ****p<0.0001, ANOVA p<0.0001. (C) Ungrouped results of OEF; ****p<0.0001, ANOVA p<0.0001.

This hemodynamic response was observed in all mice, regardless of mouse line, with a significant 88% increase in OEF (Fig 5C). In addition, this response is associated with a significant drop in the arterial and venous blood flow rates immediately after SBR (represented in Fig 4C–4F), by 25% and 52%, respectively (Fig 5A and 5B). In addition, venous flow rate continues to remain diminished until POD3 (Fig 5B). OEF, after immediate rapid increase, significantly decreased by 57% on POD3 (Fig 5C).

**Fig 6:**
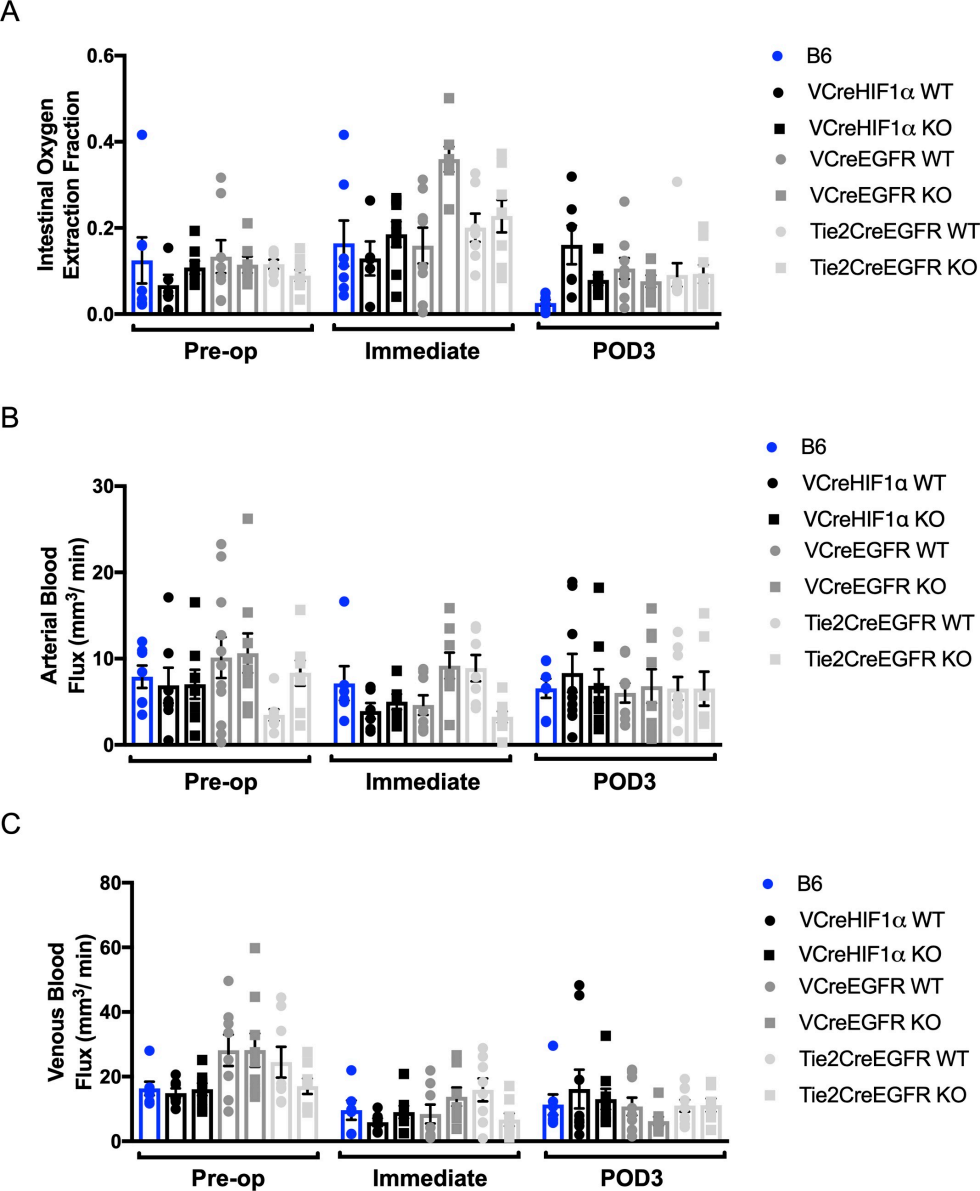
The hemodynamic response of (A) arterial blood flow, (B) venous blood flow, and (C) OEF for each operative group (n = 6–9) at pre-operative, immediate, and POD3 timepoints.

However, when divided by genotype, time has a statistically significant effect on the measured venous flux and OEF but not arterial flux. EGFR deficiency in epithelial and endothelial cells and HIF1α deficiency in epithelial cells do not affect arterial blood flow, venous blood flow, or OEF (Fig 6A–6C and Table 1).

**Table 1:** Summary of the significance of the effects of time (pre-op/immediate/POD3), genetic alteration (control/KO/WT per mouse line), and their interaction on the hemodynamics, which is evaluated by p-values calculated by 2-way ANOVA.

	Arterial flux	Venous flux	OEF
**Time**	ns	<0.0001	<0.0001
**Genotype**	ns	ns	ns

### Expression of EGFR in stromal ISEMFs is dispensable to small intestinal angiogenesis

**Fig 7:**
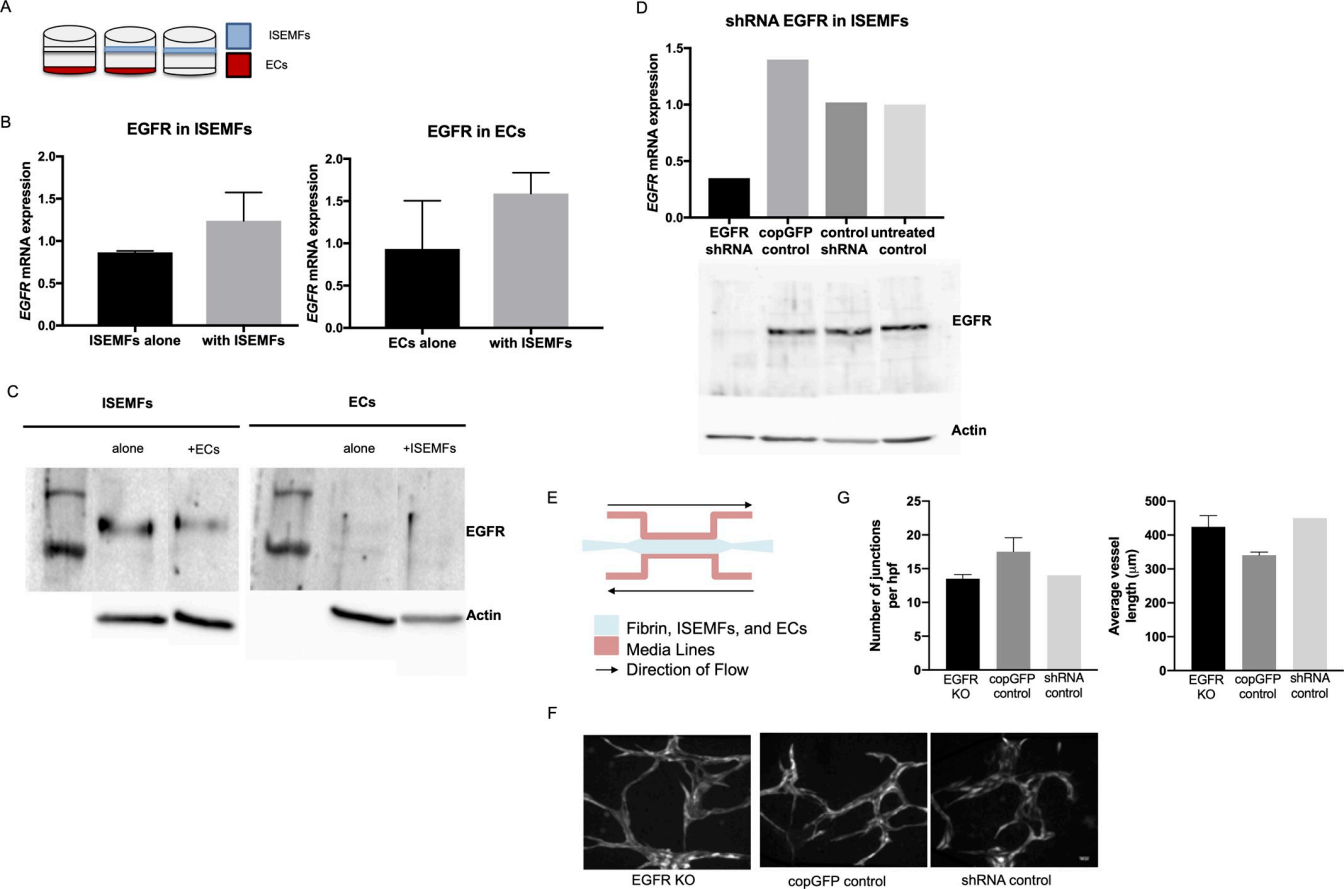
EGFR expression and knockout in ISEMFs. (A) Transwell co-culture of ECs and ISEMFs was performed as depicted. (B) After 24h in culture, RNA was isolated and quantitative RT-PCR performed for EGFR expression (n = 3 per condition). (C) Western blot for EGFR protein expression in ECs and ISEMFs cultured either alone or together. (D) Expression of EGFR mRNA (upper panel) and protein (lower panel) in ISEMFs transduced with EGFR shRNA lentiviral particles, with copGFP and shRNA lentiviral controls as indicated. (E) Schematic of microfluidic device system. (F) Representative images of vessel networks formed in microfluidic devices with fluorescent ECs and ISEMFs transduced with EGFR shRNA silencing (left), as opposed to copGFP (middle) and shRNA (right) lentiviral controls. (G) Quantitative analysis of vessel networks with number of junctions per high power field and average vessel length in microfluidic devices with fluorescent ECs and ISEMFs transduced with EGFR shRNA silencing (n = 4,3), copGFP (n = 6,3), and shRNA (n = 1,1) lentiviral controls, respectively.

Thus far, our results have demonstrated no effect on epithelial HIF1α KO or EGFR KO in either epithelium or endothelium on SBR induced angiogenesis. This led us to explore one alternative source of EGFR signaling during angiogenesis–SI stromal cells. We chose to explore this because we have previously demonstrated that ISEMFs are necessary for EC capillary development in an *ex vivo* microfluidic culture system[44]. We interrogated the role of ISEMF EGFR by performing ISEMF and EC co-culture experiments (Fig 7A). This revealed a moderate but insignificant increase in EGFR mRNA expression in both ISEMFs and ECs when cultured in the presence of one another (Fig 7B). However, we were only able to detect EGFR protein expression in ISEMFs (Fig 7C). Thus, we subsequently performed shRNA knockout of EGFR in ISEMFs to determine whether angiogenesis would still be affected. Silencing of EGFR in ISEMFs was confirmed using quantitative RT-PCR for mRNA (Fig 7D, upper panel), and western blot for protein expression (Fig 7D, lower panel). Subsequently, ISEMFs transduced with EGFR shRNA were co-cultured with fluorescently labeled ECs in microfluidic devices for 7 days. Silencing of EGFR in ISEMFs did not inhibit vessel network formation in this system, qualitatively and quantitatively (Fig 7F–7G). This led us to conclude that EGFR expression in at least three cell types (epithelium, endothelium, and ISEMFs) is dispensable to intestinal angiogenesis.

## Discussion

Massive small bowel resection results in abrupt reductions in blood flow to the remnant bowel with elevated levels of HIF1α, a compensatory increase in villus height, and corresponding angiogenesis[9, 17]. Our goal in this study was to determine if EGFR or HIF1α signaling in the epithelium or the endothelium, are required for resection-associated hemodynamic responses, intestinal adaptation, and angiogenesis.

We have previously shown through PAM, that there is a significant fall in oxygen saturation and blood flow within the remnant bowel immediately after SBR *in vivo*[16]. This was replicated in our conglomerated data, with a significant fall in arterial and venous blood flow with a corresponding increased demand in tissue oxygen extraction immediately after resection. The immediate decrease in blood flow likely results from the acute ligation of the mesenteric vasculature. Interestingly, by POD3, venous blood flow remained significantly reduced, but both arterial blood flow and oxygen saturation returned to baseline pre-operative values. Therefore, we posit that this is a hypoxic signal to stimulate angiogenesis and return of arterial blood flow to its normal preoperative state. However, this angiogenic signal does not affect the reestablishment of baseline venous blood flow by POD3. We believe that this may be due to angiogenic signals after SBR acting preferentially on arterial vessels, as it has been shown that arterial and venous endothelial cells are molecularly distinct with varying expressions of receptors and ligands involved in angiogenesis[46, 47]. The findings of arterial flow normalizing while venous blood flow remains diminished in the early postoperative state have previously been shown, implying that arterial blood flow responds more quickly to resection than venous blood flow[48].

Given the PAM study results, we focused on the attenuation of two key regulators of angiogenesis, HIF1α in the epithelium, and EGFR in the epithelium and endothelium, to determine if they could be the driver of signaling to return arterial blood flow to baseline after resection. We discovered that no genetic alteration affected arterial or venous blood flow and resulting tissue oxygen extraction by POD3. Additionally, we have shown that there is also no change in submucosal capillary density, a marker of angiogenesis, as well as adaptation, with loss of these proteins by POD7. Although prior work has found that inhibition of angiogenesis by selective blockade of salivary-derived vascular endothelial growth factor impairs adaptation, this study provides evidence that neither epithelial nor endothelial EGFR and epithelial HIF1α are dispensable to intestinal angiogenesis[49]. Endothelial cells are certainly responsive to EGFR agonists, as we previously demonstrated that direct treatment of human umbilical vein endothelial cells with EGF resulted in significantly increased expression of the proangiogenic chemokine CXCL5[50]. The contribution of CXCL5 to resection-induced intestinal angiogenesis was demonstrated using CXCL5-null mice, in which angiogenesis was blunted[51]. Although EGFR inhibition has been shown to attenuate intestinal adaptation, we have shown that inhibition in the epithelium and endothelium does not affect angiogenesis[52–54]. This suggests that angiogenesis and adaptation are uncoupled responses to massive intestinal resection.

From this data, we then hypothesized that another submucosal cell type, stromal myofibroblasts, might be contributing to angiogenesis by recovering the loss of EGFR ablation in ECs or enterocytes. Based on literature that ISEMFs are critical to intestinal cell lineage development, we hypothesized that ISEMFs integrate environmental stimuli to direct angiogenesis[55, 56]. We have shown that patient-derived ISEMFs have angiogenic properties and form a vascular network when co-cultured with ECs in microfluidic devices. Additionally, we have shown that inhibition with Erlotinib, a receptor tyrosine kinase inhibitor of EGFR, prevents vasculogenesis in these co-cultures[44]. Given these prior findings, and the fact that we have shown that EGFR was strongly expressed in ISEMFs, we used this *ex vivo* microfluidic model of human small intestine to study the effects of EGFR signaling from ISEMFs on ECs. We found that silencing of SIMF EGFR did not prevent capillary network formation, leading us to conclude either that Erlotinib may be exhibiting off-target effects to inhibit angiogenesis, independent of EGFR, or that there are other EGFR-responsive cell types that contribute to adaptive angiogenesis[57].

Despite ablating EGFR in three cell lines, including enterocytes, ECs, and ISEMFs, there was no significant impact on angiogenesis after massive SBR. A limitation of this study includes the small sample size, commonly seen in animal studies, and partial deletion of EGFR in EC cells; we only confirmed EGFR deficiency in representative samples. We cannot conclude that EGFR is dispensable to SI angiogenesis in all situations, as several groups have described that EGFR, and especially endothelial EGFR, becomes conditionally activated or expressed during times of challenge such as hypoxia, toxicity, or malignancy[58–61]. Additionally, despite showing increased intestinal expression of HIF1α after SBR, epithelial HIF1α expression was dispensable to angiogenesis by POD7[62]. We now hypothesize that circulating humoral factors after SBR, which have been shown to induce SI adaptation, are accounting for the lack of impact on angiogenesis after these selective cell line ablations[63, 64].

## Conclusion

Overall, despite the importance of EGFR in facilitating intestinal adaptation after SBR, it had no impact on angiogenesis in three cell types–enterocytes, ECs, and ISEMFs. Despite the initial increase in HIF1α expression after SBR as well as immediate increase in demand in oxygen extraction fraction via PAM, epithelial ablation of HIF1α had no impact on angiogenesis in the setting of SBS.

## Supporting information

S1 Fig (PDF)

S1 Dataset (XLSX)

## Abbreviations

SBS: Short bowel syndrome
SI: small intestine
SBR: small bowel resection
PAM: photoacoustic microscopy
HIF1α: hypoxia-inducing factor 1-alpha
EGFR: Epidermal Growth Factor Receptor
ECs: endothelial cells
pre-op: pre-operative
POD: postoperative day
IO: intraoperative
sO_2_: oxygen saturation
RBCs: red blood cells
OEF: oxygen extraction fraction
ISEMFs: intestinal subepithelial myofibroblasts
ECs: endothelial cells
WT: wildtype
KO: knockout
