# Supplementary figures and images for: EGFR in enterocytes & endothelium and HIF1α in enterocytes are dispensable for massive small bowel resection induced angiogenesis
Source: PLoS One. 2020 Sep 15;15(9):e0236964. doi: 10.1371/journal.pone.0236964 (PMC7491746; doi:10.1371/journal.pone.0236964)

Figure 7C

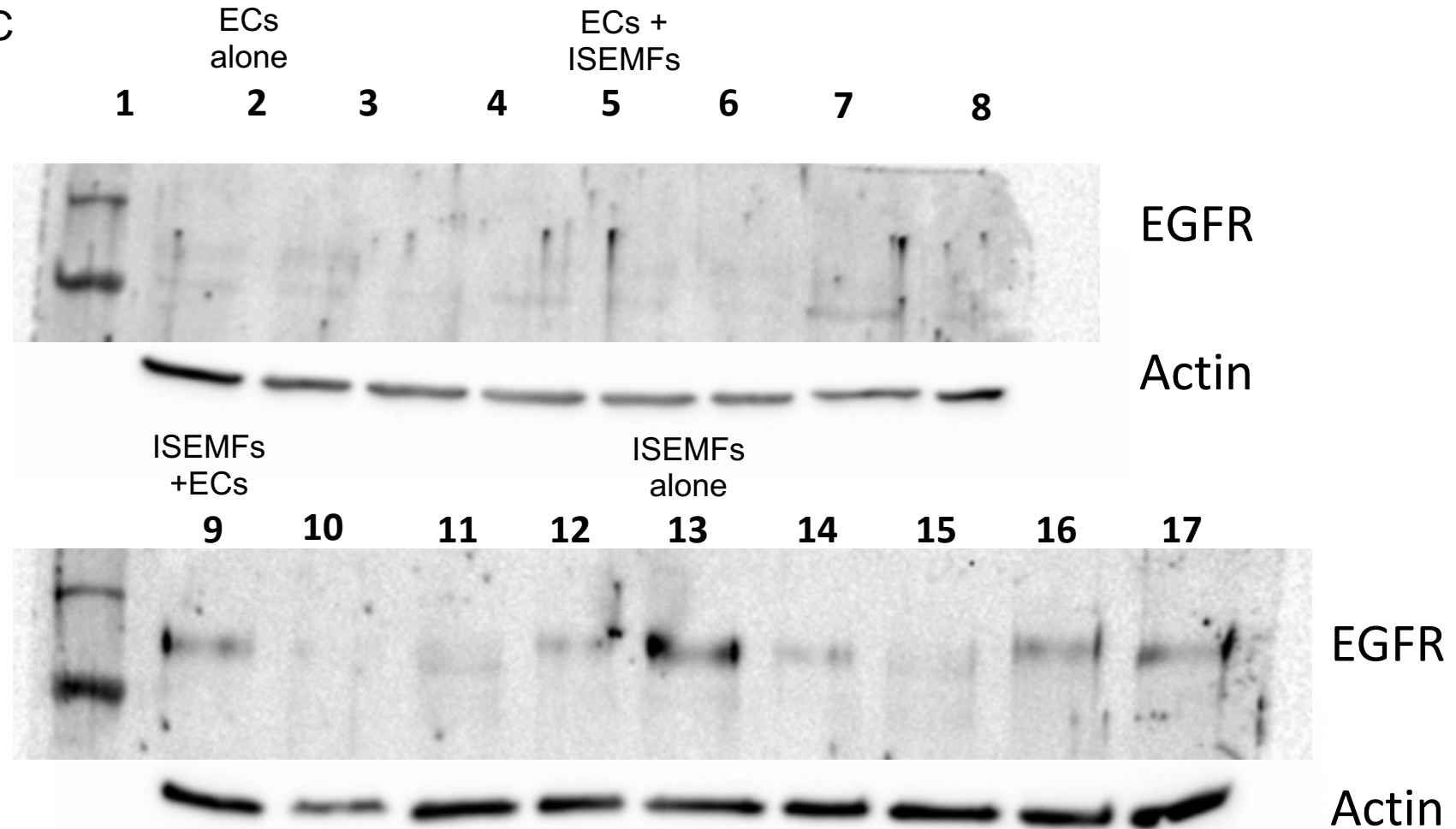

Figure 7D

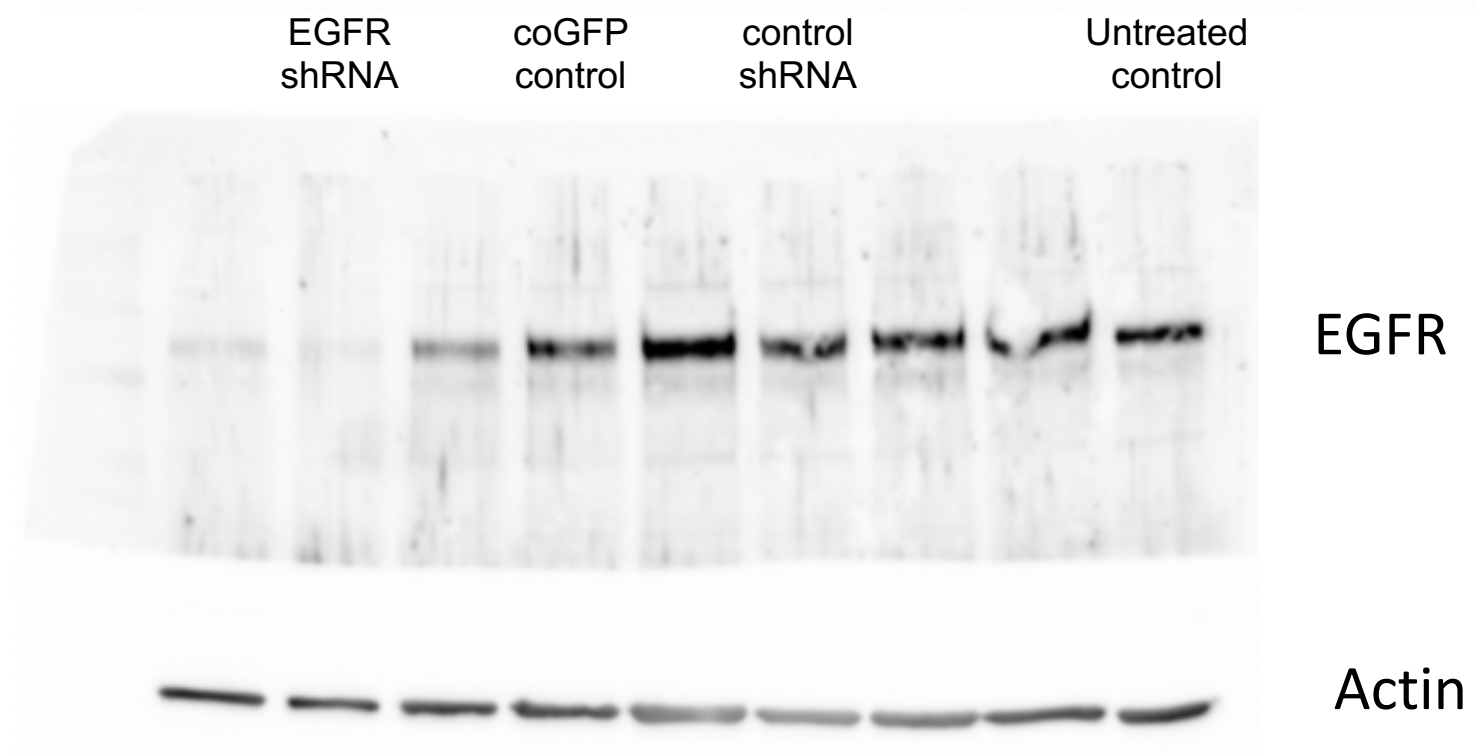

Supplement: S1 Fig — (PDF) [file pone.0236964.s001.pdf]
